# The complete mitochondrial genome of *Urocitellus undulatus* and its phylogenetic analysis

**DOI:** 10.1080/23802359.2025.2503410

**Published:** 2025-05-11

**Authors:** Zimeng Liu, Lei Chen, Yuran Pang, Xing Yang, Fengyan Zhang, Tangxin Liu, Xinhui Zhang, Yayun He, Xu He, Jiaxin Wang, Qingsong Sun

**Affiliations:** a College of Animal Science and Technology, Jilin Agricultural Science and Technology University, Jilin Jilin, China; b The Base for Control and Prevention of Plague and Brucellosis, Chinese Center for Disease Control and Prevention, BaiCheng Jilin, China; c Integrated Laboratory of Pathogenic Biology, College of Preclinical Medicine, Dali University, Dali, People’s Republic of China

**Keywords:** Mitochondrial genome, *Urocitellus undulatus*, Sciuridae, phylogeny

## Abstract

The long-tailed ground squirrel (*Urocitellus undulatus*) is primarily distributed in various regions, including Xinjiang and Heilongjiang in China, as well as in Siberia, Mongolia, the Russian Federation, and Kazakhstan. In this study, we report the complete mitochondrial genomes of *U. undulatus*. The genome consists of 16,456 base pairs, including 13 protein-coding genes, 22 tRNA genes, 2 rRNA genes, and a control region. The phylogenetic relationship indicates a closer evolutionary relationship between *U. undulatus* and *U. richardsonii*. These findings provide a foundation for the taxonomic identification, phylogenetic evolution, and mitochondrial genome research of *U. undulatus*.

## Graphical Abstract

**Figure:**
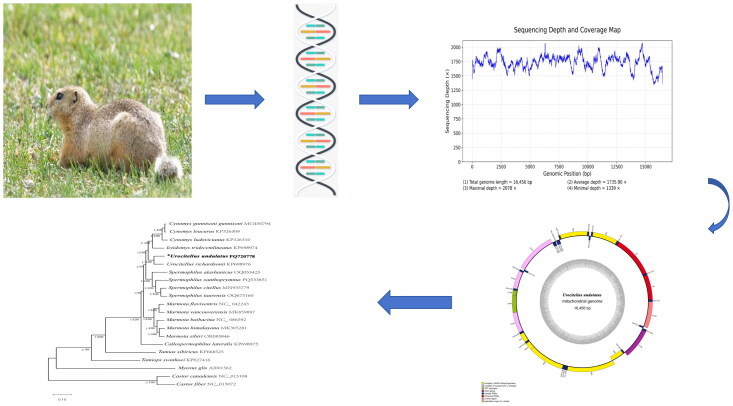

## Introduction

The long-tailed ground squirrel (*Urocitellus undulatus* Pallas, 1778) belongs to the rodent family Sciuridae, within the *Urocitellus* genus. It is distributed in various regions, including China (Xinjiang, Heilongjiang), Siberia, Mongolia, Russia, and Kazakhstan (McLean et al. 2018). These squirrels predominantly inhabit the sparse tree grasslands and vast prairie areas at the edge of the Gobi Desert. They particularly prefer areas with well-drained, loamy soils, which make it easier for them to dig their burrows (Ricankova et al. 2006; Kryštufek and Vohralík 2013). As an omnivorous species, *U. undulatus* primarily feeds on vegetation found in the grasslands, including grass, seeds, and roots. However, to obtain additional protein, they also eat insects (Smith et al. 2010). Like other species of the family Sciuridae, the burrows of *U. undulatus* are divided into resident and temporary burrows. The resident burrows are more complex in structure and differ between summer and winter, whereas the temporary burrows have simpler tunnels and are primarily used for escaping from predators.

Due to the wide distribution and large population of *U. undulatus*, it was classified as a “Least Concern” species by the International Union for Conservation of Nature (IUCN) in 2016. However, as a reservoir for many pathogens (Demina et al. 2017; Li et al. 2019a), accurate identification of *U. undulatus* is crucial. Mitochondrial genomes are commonly employed in species identification and evolutionary studies due to their conserved gene content, maternal inheritance, and compact molecular structure (Tatarenkov and Avise 2007; Gao et al. 2017). In this study, the mitochondrial genome of *U. undulatus* was determined and annotated, providing a valuable reference for future research on this species, as well as establishing a basis for the classification and evolutionary analysis of the Sciuridae family.

## Materials and methods

### Sample collection and DNA extraction

**Figure 1.**
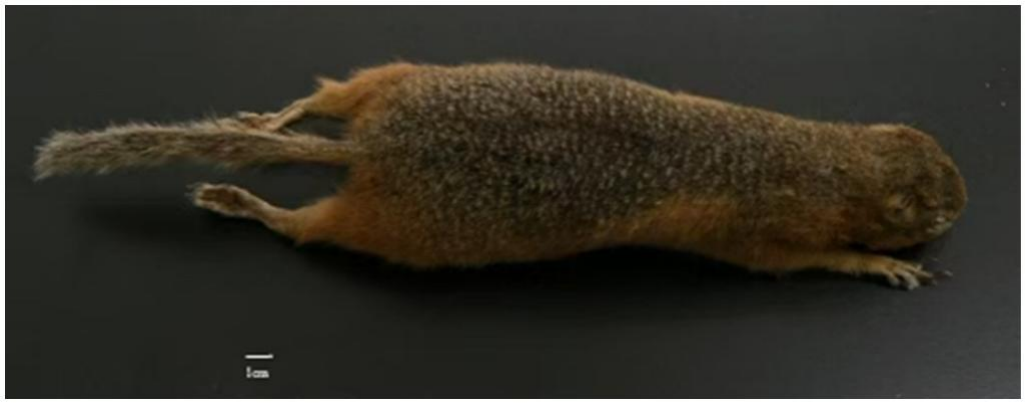
Species reference image of *Urocitellus undulatus* collected from Qinghe County, photographed by Qingsong Sun.

In June 2024, a male adult squirrel was collected as a specimen after natural death in Qinghe County (89°47′E, 45°00′N), Xinjiang Uygur Autonomous Region, China. The specimen’s dorsal fur was gray-brown with white spots, and its claws were gray-brown. Diagnostic characteristics confirmed the specimen as *U. undulatus* (Rodentia: Sciuridae) (Figure 1). After collection, the specimen was first disinfected with 75% ethanol. A small sterile muscle sample was then taken from its leg and preserved at −20 °C for further analysis. Voucher specimens have been preserved in the Dali University Biological Herbarium (voucher number DLU 240589) (URL: http://www.dali.edu.cn/jcyxy/xkpt/jcyxsyjxzx/6431.htm). Contact person: Xing Yang, yang08220013@163.com. Genomic DNA was extracted following the protocol provided by the TIANamp Genomic DNA Kit (Tiangen, Beijing, China) as recommended by the manufacturer.

### Sequence, assembly, and annotation analysis

DNA samples were processed by Harbin Botai Biological Co., Ltd. using the Illumina NovaSeq 6000 platform. Libraries were prepared with a whole-genome shotgun approach, featuring 350 bp insert fragments. Paired-end (PE) sequencing was conducted with 150 bp reads at both ends of the DNA fragments. Raw sequencing reads were first assessed for quality metrics using FastQC (Brown et al. 2017). To ensure robust downstream analyses, raw data were filtered to remove low-quality sequences, yielding high-quality clean reads. The high-quality sequencing data were assembled using SPAdes v3.14.1 (Bankevich et al. 2012), with multiple k-mer values applied to further optimize the assembly. Mitochondrial protein-coding genes (PCGs), transfer RNA genes (tRNAs), and rRNA genes (rRNAs) were annotated using MITOS (Bernt et al. 2013). Genome architecture was visualized as a circular map *via* Organellar Genome DRAW v1.2 (Greiner et al. 2019). The AT and GC content of each gene were analyzed using DNAStar software, and the AT-Skew and GC-Skew are computed using these equations: AT skew = (A − T)/(A + T) and GC skew = (G − C)/(G + C) (Perna and Kocher 1995).

### Phylogenetic analysis

Phylogenetic relationships between *U. undulatus* and other closely related species were inferred using maximum likelihood (ML) and Bayesian inference (BI) methods. Downloaded the 13 PCGs from 21 species from NCBI, followed by sequence trimming and alignment using MEGA v 11.0. The ML method was executed in MEGA v 11.0 with 1,000 bootstrap replicates (Tamura et al. 2021). The most appropriate model was inferred to be GTR+G + I based on ModelFinder. The BI analysis was performed using MrBayes version 3.2.7, involving 1,000,000 iterations, with a sample taken every 1000 generations, and the initial 25% of the trees were excluded as burn-in (Ronquist and Huelsenbeck 2003). The phylogenetic tree was viewed and modified using FigTree software.

## Results

**Figure 2.**
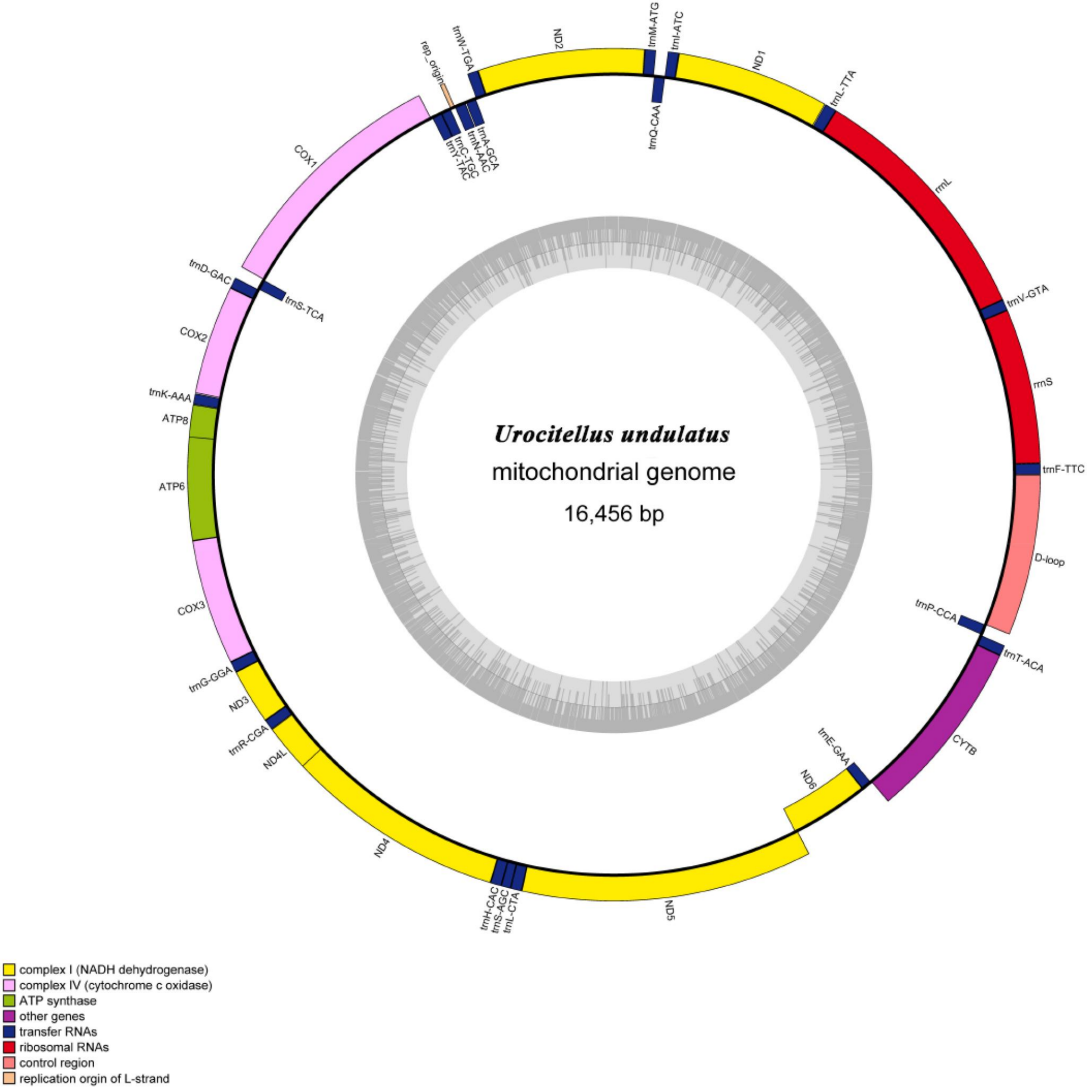
The mitochondrial genome map of *U. undulatus.*

The mitochondrial genome of *U. undulatus* spans 16,456 bp in total length (GenBank accession number: PQ720778) and is illustrated in Figure 2, with a mean coverage of trimmed sequencing data at 1735.90× (Figure S1). The mitogenome comprises the typical 37 genes found in animals, consisting of 13 PCGs, 2 rRNAs, 22 tRNAs, plus a control region (Figure 2). Nucleotide composition was adenine (32.33%), thymine (30.92%), guanine (12.64%), and cytosine (24.11%). The mitogenome consists of 63.25% A + T content and 36.75% G + C content. In the analysis of AT-skew and GC-skew for the entire mitochondrial genome, the obtained values were 0.022 and −0.312, respectively (Table S1). Two rRNA genes (*rrnL* and *rrnS*), 14 tRNAs (*trnV*, *trnF*, *trnL1*, *trnM, trnD, trnW*, *trnI*, *trnR*, *trnG*, *trnK*, *trnH*, *trnS*, *trnL,* and *trnT*) genes and 11 PCGs (*nad1-5, cox1-3*, *atp6, atp8*, *cob* and *nad4L*) were situated on the majority stand. The minority stand contained a PCG (*nad6*), and eight tRNA genes (*trnC*, *trnY*, *trnN*, *trnQ*, *trnA*, *trnS2*, *trnP* and *trnE*) (Table S2). The 13 PCGs had a sequence length of 11,398 bp, comprising 69.26% of the entire mitochondrial genome. Nucleotide composition was adenine (30.88%), thymine (32.47%), guanine (12.44%), and cytosine (24.71%). The PCGs consist of 62.85% A + T content and 37.15% G + C content. Start codons for all PCGs were ATN, but different protein coding genes correspond to different termination codons; of which 6 stop codons were TAA (*cox1*, *cox2*, *atp8*, *atp6*, *nad4L*, and *nad5*), 2 stop codons were AGA (*nad6* and *cob*), 2 incomplete stop codons were TA(A) (*nad1* and *nad3*) and 3 incomplete stop codons were T(AA) (*nad2*, *cox3* and *nad4*). tRNA lengths vary from 74 bp to 59 bp, with *trnL-TTA* being the extended sequence at 74 base pairs while *trnS-AGC* is the shortest at 59 base pairs. The control region, spanning 1,006 bp, is positioned between *trnF* and *trnP*. The *rrn*L and *rrn*S are located on either side of *trnV,* with lengths of 1,572 bp and 967 bp, respectively.

**Figure 3.**
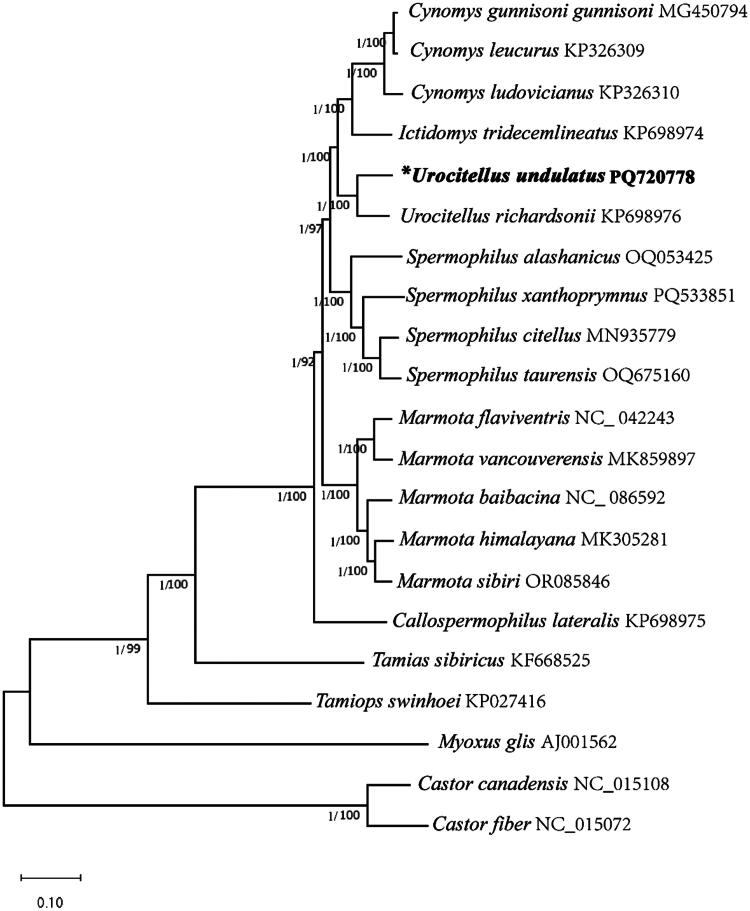
Phylogenetic relationships of the Sciuridae family inferred using BI and ML analyses based on 13 PCGs of mitogenomes. The following sequences were used: *Cynomys gunnisoni gunnisoni* (Streich et al. 2019), *Cynomys leucurus* and *Cynomys ludovicianus* (Li et al. 2016), *Ictidomys tridecemlineatus* (Zhang et al. 2016b), *Urocitellus undulatus* (this study), *Urocitellus richardsonii* and *Callospermophilus lateralis* (Zhang et al. 2016a), *Spermophilus alashanicus* (Zhao et al. 2024), *Sper*moph*ilus xanthoprymnus* (Unpublished), *Spermophilus citellus* (Krystufek et al. 2009), *Spermophilus taurensis* (Matrosova et al. 2023), *Marmota flaviventris* (Unpublished), *Marmota vancouverensis* (Hao and Cao 2019), *Marmota baibacina* (Unpublished), *Marmota himalayana* (Li et al. 2019b), *Marmota sibiri* (Unpublished), *Tamias sibiricus* (Yoon et al. 2015), *Tamiops swinhoei* (Xu et al. 2016), *Myoxus glis* (Xu et al. 2016), *Castor canadensis* and *Castor fiber* (Horn et al. 2011).

Phylogenetic trees were constructed by concatenating 13 protein gene sequences from 21 species and using ML and BI methods (Figure 3). *Myoxus glis* (Gliridae), *Castor canadensis*, and *C. fiber* (Castoridae), and as outgroups to confirm the phylogenetic relationship of Sciuridae. In the BI and ML trees, *U. undulatus* was sister to that of *U. richardsonii* with high bootstrap support (1.00 in BI and 100% in ML). This phylogenetic analysis provides useful genetic resources for revealing the evolution of the family Sciuridae.

## Discussion and conclusion

In this study, the entire mitochondrial genome of *U. undulatus* was sequenced. Consistent with most metazoan mitochondrial genomes, it contains 37 functional genes (Boore 1999). However, some previously published animal mitochondrial genomes show differences from other metazoan genomes, as their mitochondrial genomes lack the *atp8* gene (Yamasaki et al. 2012; Hao et al. 2024). The length and gene arrangement of the *U. undulatus* mitochondrial genome are consistent with those of other Sciuridae family species. All protein-coding genes utilize the standard mitochondrial start codons (ATG, ATA, ATT), and it has been observed that the genes *nad1*, *nad2*, *nad3*, *nad4*, and *cox3* possess incomplete stop codons. This phenomenon has also been found in other Sciuridae family species, such as *S. taurensis* and *M. himalayana* (Li et al. 2019b; Matrosova et al. 2023). However, these incomplete stop codons will eventually form complete stop codons through post-transcriptional polyadenylation modifications (Beckenbach and Stewart 2009; Chen et al. 2012; Donath et al. 2019). The content of AT is higher than that of GC, which is common in mitochondrial studies of some animals (Hassanin et al. 2005).

Two methods were utilized to construct the phylogenetic tree of the Sciuridae family, and the species within the family clustered together with high confidence. The results show that *U. undulatus* and *U. richardsonii* are closely related, with species within the same genus (*Urocitellus*) clustering together. This study fills the gap in the mitochondrial genome of *U. undulatus*, lays the foundation for the systematics and evolution of the genus *Urocitellus,* providing valuable resources for the species evolution, taxonomy, species identification, and population genetics research of the Sciuridae family.

## Supplementary Material

Supplementary Figure 1.png

Supplementary Table 1.docx

Supplementary Table 2.docx

Biographical note.docx

## Data Availability

The data supporting the findings of this investigation may be found at https://www.ncbi.nlm.nih.gov/ under the reference number PQ720778. The associated BioProject, SRA, and Bio-Sample numbers are PRJNA1216044, SRR32134887, and SAMN46424648, respectively.
