# Supplementary figures and images for: The complete mitochondrial genome of Urocitellus undulatus and its phylogenetic analysis
Source: Mitochondrial DNA B Resour. 2025 May 11;10(6):453–8. doi: 10.1080/23802359.2025.2503410 (PMC12077428; doi:10.1080/23802359.2025.2503410)

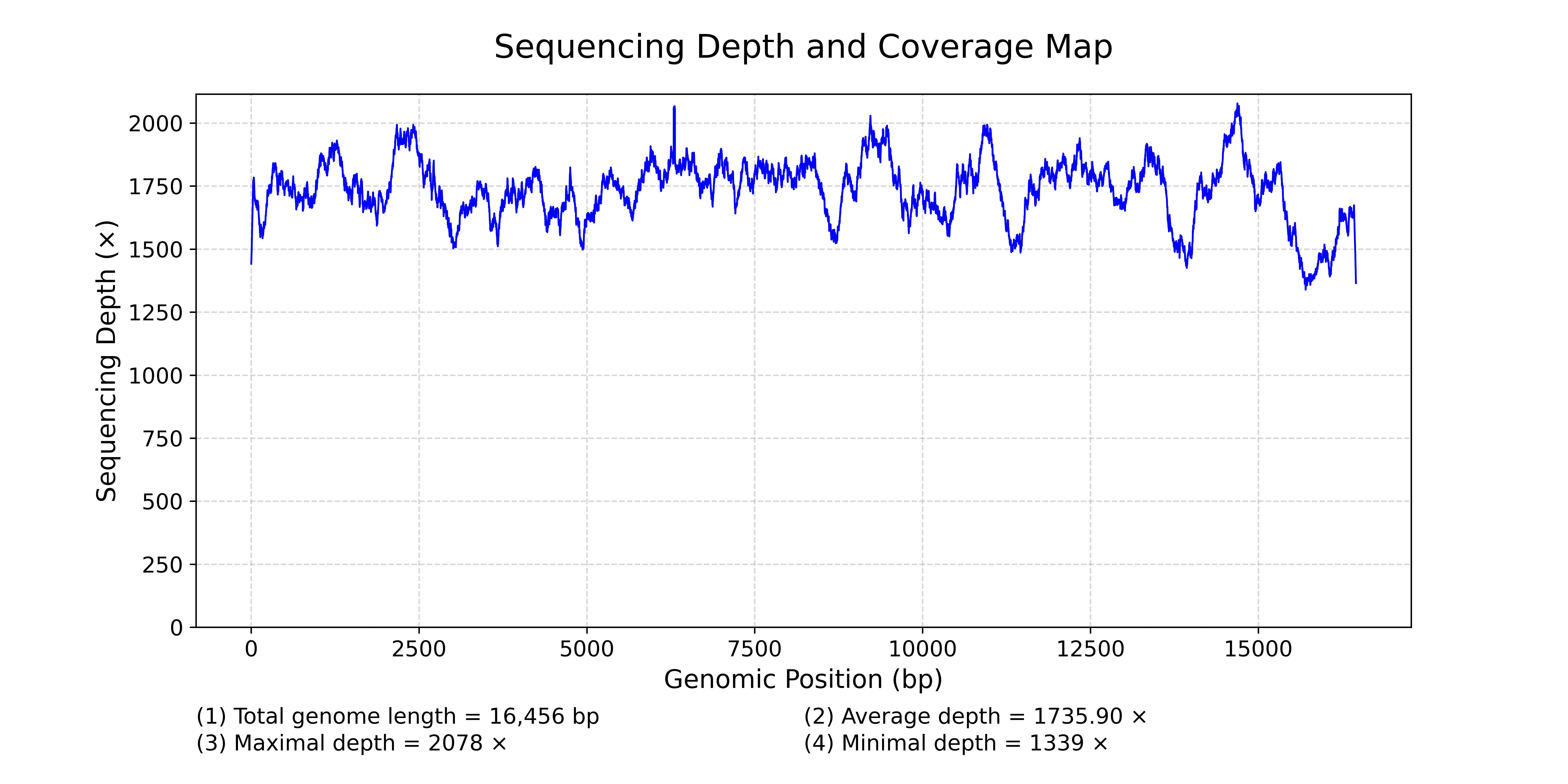

Supplement: Supplementary Figure 1.png [file TMDN_A_2503410_SM5476.png]
