# Supplementary material for: The complete mitochondrial genome of Urocitellus undulatus and its phylogenetic analysis
Source: Mitochondrial DNA B Resour. 2025 May 11;10(6):453–8. doi: 10.1080/23802359.2025.2503410 (PMC12077428; doi:10.1080/23802359.2025.2503410)
Supplement: Supplementary Table 1.docx [file TMDN_A_2503410_SM5475.docx]

| Feature | Size (bp) | A% | G% | T% | C% | A+T% | AT Skew | GC Skew |
| --- | --- | --- | --- | --- | --- | --- | --- | --- |
| Mitogenome | 16,456 | 32.33 | 12.64 | 30.92 | 24.11 | 63.25 | 0.022 | -0.312 |
| PCGs | 11,398 | 30.88 | 12.44 | 32.47 | 24.71 | 62.85 | -0.025 | -0.330 |
| tRNAs | 1512 | 33.99 | 18.06 | 32.21 | 15.74 | 66.20 | 0.027 | 0.069 |
| rRNAs | 2539 | 36.39 | 16.86 | 26.55 | 20.20 | 62.94 | 0.156 | -0.090 |
| D-loop | 1006 | 31.61 | 12.13 | 32.80 | 23.46 | 64.41 | -0.018 | -0.318 |
| *Cox3* | 784 | 27.81 | 14.80 | 30.74 | 26.66 | 58.55 | -0.050 | -0.286 |
| *Cob* | 1140 | 28.60 | 12.63 | 32.63 | 26.14 | 61.23 | -0.066 | -0.348 |
| *Nad4l* | 297 | 27.95 | 10.77 | 38.72 | 22.56 | 66.67 | -0.162 | -0.354 |
| *Nad4* | 1378 | 32.37 | 9.51 | 32.66 | 25.47 | 65.02 | -0.004 | -0.456 |
| *Atp6* | 681 | 30.54 | 11.01 | 32.31 | 26.14 | 62.85 | -0.028 | -0.407 |
| *Nad2* | 1042 | 34.84 | 8.35 | 29.56 | 27.26 | 64.40 | 0.082 | -0.531 |
| *Nad1* | 956 | 31.80 | 10.77 | 31.77 | 26.26 | 62.97 | 0.000 | -0.418 |
| *Nad3* | 347 | 27.38 | 12.68 | 33.72 | 26.22 | 61.10 | -0.104 | -0.348 |
| *Cox1* | 1542 | 27.63 | 16.15 | 34.05 | 22.18 | 61.67 | -0.104 | -0.157 |
| *Cox2* | 684 | 32.60 | 12.28 | 31.43 | 23.68 | 64.04 | 0.018 | -0.317 |
| *Nad6* | 525 | 24.38 | 28.57 | 40.57 | 6.48 | 64.95 | -0.249 | 0.630 |
| *Nad5* | 1818 | 31.24 | 10.45 | 31.02 | 27.28 | 62.27 | 0.004 | -0.446 |
| *Atp8* | 204 | 36.76 | 6.37 | 30.88 | 25.98 | 67.65 | 0.087 | -0.606 |
| *rrnL* | 1572 | 37.09 | 16.35 | 27.35 | 19.21 | 64.44 | 0.151 | -0.080 |
| *rrnS* | 967 | 36.26 | 17.68 | 25.23 | 21.82 | 60.50 | 0.182 | -0.105 |
