# Supplementary material for: The complete mitochondrial genome of Urocitellus undulatus and its phylogenetic analysis
Source: Mitochondrial DNA B Resour. 2025 May 11;10(6):453–8. doi: 10.1080/23802359.2025.2503410 (PMC12077428; doi:10.1080/23802359.2025.2503410)
Supplement: Supplementary Table 2.docx [file TMDN_A_2503410_SM5474.docx]

| Gene | Strand | Location | Size (bp) | Start codon | Stop codon |
| --- | --- | --- | --- | --- | --- |
| *trnA^Phe^* | J | 1-70 | 70 |  |  |
| *rrnS* | J | 71-1037 | 967 |  |  |
| *trnA^Val^* | J | 1038-1107 | 70 |  |  |
| *rrnL* | J | 1108-2679 | 1572 |  |  |
| *trnA^Leu（TTA）^* | J | 2680-2753 | 74 |  |  |
| *nad1* | J | 2757-3712 | 956 | ATG | TA(A) |
| *trnA^Ile^* | J | 3713-3781 | 69 |  |  |
| *trnA^Gln^* | N | 3850-3779 | 72 |  |  |
| *trnA^Met^* | J | 3854-3922 | 69 |  |  |
| *nad2* | J | 3923-4964 | 1042 | ATT | T(AA) |
| *trnA^Trp^* | J | 4965-5032 | 68 |  |  |
| *trnA^Ala^* | N | 5104-5036 | 69 |  |  |
| *trnA^Asn^* | N | 5182-5110 | 73 |  |  |
| RP | J | 5183-5213 | 31 |  |  |
| *trnA^Cys^* | N | 5280-5214 | 67 |  |  |
| *trnA^Tyr^* | N | 5346-5281 | 66 |  |  |
| *cox1* | J | 5355-6896 | 1542 | ATG | TAA |
| *trnA^Ser（TCA）^* | N | 6967-6899 | 69 |  |  |
| *trnA^Asp^* | J | 6971-7039 | 69 |  |  |
| *cox2* | J | 7040-7723 | 684 | ATG | TAA |
| *trnA^Lys^* | J | 7727-7793 | 67 |  |  |
| *atp8* | J | 7795-7998 | 204 | ATG | TAA |
| *atp6* | J | 7956-8636 | 681 | ATG | TAA |
| *cox3* | J | 8636-9419 | 784 | ATG | T(AA) |
| *trnA^Gly^* | J | 9420-9489 | 70 |  |  |
| *nad3* | J | 9490-9836 | 347 | ATA | TA(A) |
| *trnA^Arg^* | J | 9837-9903 | 67 |  |  |
| *nad4l* | J | 9905-10201 | 297 | ATG | TAA |
| *nad4* | J | 10195-11572 | 1378 | ATG | T(AA) |
| *trnA^His^* | J | 11573-11641 | 69 |  |  |
| *trnA^Ser（AGC）^* | J | 11642-11700 | 59 |  |  |
| *trnA^Leu（CTA）^* | J | 11701-11770 | 70 |  |  |
| *nad5* | J | 11771-13588 | 1818 | ATA | TAA |
| *nad6* | N | 14096-13572 | 525 | ATG | AGA |
| *trnA^Glu^* | N | 14165-14097 | 69 |  |  |
| *cob* | J | 14170-15309 | 1140 | ATG | AGA |
| *trnA^Thr^* | J | 15310-15376 | 67 |  |  |
| *trnA^Pro^* | N | 15450-15382 | 69 |  |  |
| D-loop |  | 15451-16456 | 1006 |  |  |
