# Supplementary material for: The complete mitochondrial genome of Urocitellus undulatus and its phylogenetic analysis
Source: Mitochondrial DNA B Resour. 2025 May 11;10(6):453–8. doi: 10.1080/23802359.2025.2503410 (PMC12077428; doi:10.1080/23802359.2025.2503410)
Supplement: Biographical note.docx [file TMDN_A_2503410_SM5473.docx]

Zimeng Liu, Lei Chen, Yuran Pang, Xing Yang, Fengyan Zhang, Tangxin Liu, Xinhui Zhang, Yayun He, Xu He, and Jiaxin Wang primarily focus on the mitochondrial genome and taxonomy research of vertebrates, ticks, and mosquitoes, while also paying attention to zoonotic parasitic diseases in animals and humans. Dr. Sun Qingsong, PhD, is an associate professor at the College of Animal Science and Technology, Jilin University of Agricultural Science and Technology, and serves as the head of the veterinary department. His research primarily focuses on animal and zoonotic parasitic diseases. Dr. Sun has been actively involved in the academic community, delivering presentations at international health conferences. From 2021 to 2023, he led several scientific research projects and co-authored numerous academic papers spanning various domains of veterinary and animal medicine, demonstrating his profound expertise in these areas. Additionally, in 2024, Dr. Sun presented a report titled "Investigation of Food-Borne Parasite Infections in Common Freshwater Fish in the Songhua River Basin of Jilin City" at the International Conference on Total Health. This presentation highlighted his research findings on the infection and control of food-borne zoonotic parasites in the Songhua River Basin.
